# The relationship between individual differences in gray matter volume and religiosity and mystical experiences: A preregistered voxel‐based morphometry study

**DOI:** 10.1111/ejn.14563

**Published:** 2019-09-23

**Authors:** Michiel van Elk, Lukas Snoek

**Affiliations:** ^1^ Department of Psychology University of Amsterdam Amsterdam The Netherlands; ^2^ Amsterdam Brain and Cognition Center University of Amsterdam Amsterdam The Netherlands; ^3^ Spinoza Center for Neuroimaging Royal Netherlands Academy of Arts and Sciences Amsterdam The Netherlands

**Keywords:** gray matter volume, mystical experience, religiosity, structural brain differences, voxel‐based morphometry

## Abstract

The neural substrates of religious belief and experience are an intriguing though contentious topic. Here, we had the unique opportunity to establish the relation between validated measures of religiosity and gray matter volume in a large sample of participants (*N* = 211). In this registered report, we conducted a confirmatory voxel‐based morphometry analysis to test three central hypotheses regarding the relationship between religiosity and mystical experiences and gray matter volume. The preregisterered hypotheses, analysis plan, preprocessing and analysis code and statistical brain maps are all available from online repositories. By using a region‐of‐interest analysis, we found no evidence that religiosity is associated with a reduced volume of the orbito‐frontal cortex and changes in the structure of the bilateral inferior parietal lobes. Neither did we find support for the notion that mystical experiences are associated with a reduced volume of the hippocampus, the right middle temporal gyrus or with the inferior parietal lobes. A whole‐brain analysis furthermore indicated that no structural brain differences were found in association with religiosity and mystical experiences. We believe that the search for the neural correlates of religious beliefs and experiences should therefore shift focus from studying structural brain differences to a functional and multivariate approach.

## Graphical abstract

In this registered report, we conducted a confirmatory analysis of the relation between gray matter volume and religiosity and mystical experiences. Overall, we found no evidence for the notion that structural changes in the bilateral inferior parietal lobes, the right middle temporal gyrus and the hippocampus are related to religiosity or mystical experiences.

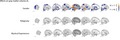

**Abbreviations**
DWI: diffusion‐weighted imaging
GLM: general linear model
IPL: inferior parietal lobe
MRI: magnetic resonance imaging
MTL: middle temporal lobe
OFC: orbitofrontal cortex
ROI: region‐of‐interest
SPL: superior parietal lobe
ToM: theory of mind
VBM: voxel‐based morphometry
VMPC: ventromedial prefrontal cortex

## 1 INTRODUCTION

In the early 2000s, several newspapers headlined a study that had found the God‐spot—a brain region that could be considered the basis of the widespread belief in an omniscient omnipresent and powerful being. This news was based on pioneering work by Andrew Newberg, who identified the neural correlates of the unitary peak experience of monks (Newberg, Alavi, et al., 2001; Newberg & Iversen, 2003). One of their key findings was that the superior parietal lobe (SPL)—a brain region that has been associated with spatial attention and temporal processing—showed a reduced activity during meditative peak experiences compared to baseline. This finding made sense in light of the phenomenological reports that often referred to feelings of a loss of sense of space and time and the awareness of a presence that was bigger than the self. These initial results inspired many neuroscientists, philosophers and theologians to reflect on the potential implications. While some argued that these brain regions could be considered a mechanism to perceive ultimate reality (Beauregard & O'Leary, 2007; Newberg, d'Aquili, & Rause, 2001), other researchers gave a more reductionist interpretation according to which religious belief and mystical experience could be considered a by‐product of the way our brains evolved (Boyer, 2003). In this manuscript, we define religiosity as the belief in an invisible supernatural agent (i.e., God) that is typically based on tradition (as united in a community of believers) and is manifested by overt behavior such as visiting a church or religious meeting and praying on a daily basis. Mystical experiences are characterized by a reduced awareness of the self, the loss of sense of space and time and the feeling of a strong connection with the surrounding world (Piedmont, 1999).

The debate on the neural correlates of religious belief and mystical experience has been fueled by other studies that provided more in‐depth insight in the brain mechanisms at play in religion. For instance, the observation that religious participants recruit brain areas involved in social cognition during prayer (Schjoedt, Stdkilde‐Jorgensen, Geertz, & Roepstorff, 2009) has led to an impressive literature on the role of hypermentalizing as a cognitive bias predisposing people to become religious (for recent critical review, see: Maij, van Harreveld, et al., 2017). Similarly, the observation that religious believers show a reduced brain response to errors (Inzlicht, McGregor, Hirsh, & Nash, 2009; Inzlicht & Tullett, 2010) has led to the idea that reduced error monitoring and prefrontal cortex functioning could be associated with the acceptance of religious ideas. In line with this suggestion, it has been found that patients with damage to the orbitofrontal cortex (OFC) have a higher likelihood of having encountered a mystical experience (Cristofori et al., 2016). Thus, the initial steps toward unraveling the neural substrates of religiosity appear promising.

At the same time, the neuroscientific study of religion has been haunted by a lack of methodological rigor (Schjoedt, 2009). Many studies suffer from small sample sizes, a lack of well‐validated tasks, and conceptual confusion about the constructs that are measured. As a consequence, it remains unclear to what extent theories about the neural substrates underlying religiosity are actually supported by the data (van Elk & Aleman, 2017). For instance, although several studies have suggested the involvement of structural temporal lobe abnormalities in religiosity, the findings are inconclusive: on the one hand, temporal lobe atrophy has been associated with increased religiosity by using a region‐of‐interest (ROI) analysis (Chan et al., 2009; Owen, Hayward, Koenig, Steffens, & Payne, 2011), while another study found that higher religiosity was associated with an increased volume of the temporal lobe, also by using an ROI voxel‐based morphometry (VBM) analysis (Kapogiannis, Barbey, Su, Krueger, & Grafman, 2009). Similarly, whereas several neuropsychological lesion‐based studies have shown that damage to the inferior parietal lobe (IPL) is associated with increased spirituality (Johnstone, Bodling, Cohen, Christ, & Wegrzyn, 2012; Johnstone & Glass, 2008; Johnstone et al., 2014; Urgesi, Aglioti, Skrap, & Fabbro, 2010), another VBM study found that an increased IPL volume was associated with higher spirituality (Van Schuerbeek, Baeken, De Raedt, De Mey, & Luypaert, 2011). Thus, the debate on the precise neural mechanisms involved in religiosity is far from settled.

In the present registered report, we had the unique opportunity to assess the relation between well‐validated measurements of religiosity and structural brain differences in a high‐powered (*N* = 224) study. This allowed us to empirically test some of the most prominent hypotheses that have been put forward regarding the neurocognitive basis of religiosity. The MRI and religiosity data for this project were already collected as part of a larger collaborative research project, but had not been analyzed in conjunction. Our religiosity scale included questions related to religious beliefs and practices. These questions have been used before in previous studies on religious beliefs and the relation with mentalizing and agency detection (Maij, van Harreveld et al., 2017; van Elk, Rutjens, & van Harreveld, 2017). We also included questions about mystical experiences, including key items taken from the mysticism scale (Hood, 1975) and the Tellegen absorption scale (Tellegen & Atkinson, 1974). By using structural brain scans and voxel‐based morphometry (Ashburner & Friston, 2000), we investigated whether increased religiosity is associated with structural differences in gray matter volume, both in a confirmatory approach using ROI analyses of brain regions suggested by the literature as well using a whole‐brain analysis. Given the large number of participants in our study, we were able to draw more robust and precise inferences about the relation between religiosity and gray and white matter volume than in previous studies (Cremers, Wager, & Yarkoni, 2017).

The specific hypotheses that we tested were based on a review of the existing literature on the neurocognitive mechanisms involved in religion and spirituality (for detailed review, see: van Elk & Aleman, 2017).

First, we tested whether a reduced volume of the bilateral orbitofrontal cortex is associated with a stronger endorsement of religious beliefs. This hypothesis follows from the theoretical framework of predictive processing (van Elk & Aleman, 2017), as well as from the cognitive resource depletion model (Schjoedt et al., 2013). Central to these theories is the notion that a process of reduced error monitoring is at the basis of willingness to accept and believe religious doctrines. Some neuropsychological studies have indeed shown that fronto‐temporal dementia and atrophy of the OFC is associated with changes in religiosity (Hayward, Owen, Koenig, Steffens, & Payne, 2011; Miller, Mychack, Seeley, Rosen, & Boone, 2001). One study found in a small subset of patients with fronto‐temporal dementia that some of these patients experienced significant changes in their personality, including an increased interest in religiosity (Miller et al., 2001). In a longitudinal study using structural brain data from 302 participants, it was found that life‐changing religious experiences were associated with a reduction in atrophy of the left OFC (Hayward et al., 2011). In contrast, in the same study more frequent participation in public religious worship was associated with a stronger atrophy of the left OFC—thereby painting a more complicated picture of the relationship between the frontal lobes and religiosity. In a small study involving data from 40 participants, it was found that increased fear of God was associated with a reduced volume of the left OFC (Kapogiannis, Barbey, Su, Krueger, et al., 2009). And a clinical study involving data from 103 participants at low or high risk for depression found that increased importance of religion and spirituality were associated with increased cortical thickness of the mesial frontal lobe (Miller et al., 2014). A study with data from 116 patients with traumatic brain injury found that lesions to the dorsolateral prefrontal cortex and the middle/superior temporal cortex were associated with increased mysticism (Cristofori et al., 2016). Similarly, it was found in 119 patients with traumatic brain injury that lesions of the ventromedial prefrontal cortex (VMPFC, which is anatomically synonymous with the OFC; Phillips, MacPherson, & Della Sala, 2002) were associated with an increase in religious fundamentalism (Zhong, Cristofori, Bulbulia, Krueger, & Grafman, 2017). Finally, a study using data from 40 participants with and without non‐clinical psychosis also found that increased intrinsic religiosity was associated with a reduced volume of the OFC (Pelletier‐Baldelli et al., 2014).

Functional brain imaging studies corroborate the notion that changes in prefrontal cortex functioning are associated with an increased acceptance of religious ideas. It has been found for instance that believers compared to skeptics show a reduced neural response to errors—which was localized to the anterior cingulate cortex (Inzlicht & Tullett, 2010; Inzlicht et al., 2009). Furthermore, it has been found that paranormal believers compared to skeptics showed a reduced activation of the right inferior frontal gyrus when inferring meaning in random pictures (Lindeman, Svedholm, Riekki, Raij, & Hari, 2013) and that religious believers compared to skeptics showed a stronger reduction in the medial and dorsolateral prefrontal cortex when listening to a prayer by a charismatic faith healer (Schjoedt, Stodkilde‐Jorgensen, Geertz, Lund, & Roepstorff, 2011). On the other hand, it has also been found that personalized prayer to God by charismatic Christians, activates the medial prefrontal cortex (MPFC)—which is considered to be part of the theory‐of‐mind‐network (Schjoedt et al., 2009). Similarly, reflecting on God's perceived level of involvement in the world has also been associated with an increased activation of the MPFC (Kapogiannis, Barbey, Su, Zamboni, et al., 2009). However, the apparent inconsistency between these findings is probably related to differences in the experimental paradigms that were used to study religiosity (i.e., prayer and reflection on traits by definition activate the theory‐of‐mind‐network). We should also bear in mind that there is not a one‐to‐one correspondence between changes in structural brain volume and functional brain data. In fact, network analysis approaches of functional brain data (e.g., by using functional or effective connectivity) may be better suited for capturing the cognitive processes underlying religiosity and mystical experience—as they tap more directly into the efficiency by which neural networks process information (Bullmore & Sporns, 2009).

Thus—although there are variable and conflicting findings—overall these studies suggest that a reduced volume of the frontal cortex—most notably the OFC is associated with an increase in religiosity. This leads to our first hypothesis that reduced volume in the OFC is associated with an increase in religious beliefs.

Second, traditionally, abnormalities in temporal lobe anatomy or function, for example, as observed in patients with temporal lobe epilepsy, have been associated with increased religiosity (for historical overview, see: Devinsky & Lai, 2008). It has been reported that patients with temporal lobe epilepsy can have profound religious experiences, which have been attributed to spontaneous epileptic spikes in temporal brain areas (Joseph, 2001; Saver & Rabin, 1997). For instance, a patient suffering from temporal epileptic seizures reported a conversion experience and receiving messianic messages (Arzy & Schurr, 2016). Furthermore, as discussed above, structural changes in the temporal lobe, for example, due to atrophy, have also been associated with an increase in religiosity (Chan et al., 2009; Owen et al., 2011). Already in an early study involving structural brain scans from 33 epilepsy patients, a negative relation was found between increased religiosity and the volume of the right hippocampus (Wuerfel et al., 2004). Furthermore, some patients with right temporal lobe atrophy—next to experiencing the usual symptoms associated with temporal lobe atrophy, such as semantic dementia—showed hyperreligiosity as well (Chan et al., 2009). In another study, using neuroanatomical data from 268 adults it was found that having had a life‐changing religious experience was associated with a stronger atrophy of the hippocampus, as shown by using a VBM ROI analysis (Owen et al., 2011). In a dataset from 80 healthy volunteers, increases in the character trait of self‐transcendence have been associated with an increased volume of the middle temporal gyrus, as well as the inferior parietal gyrus (Van Schuerbeek et al., 2011). Similarly, data from a study with 42 healthy older adults showed that higher scores on the personality trait of self‐transcendence were associated with a reduced volume of the left fronto‐temporal and parieto‐temporal cortex (Kaasinen, Maguire, Kurki, Bruck, & Rinne, 2005).

Together these findings suggest that temporal lobe regions may be specifically involved in the experiential aspects of religiosity, such as mystical experiences and feelings of self‐transcendence (Grill‐Spector & Malach, 2004). Thus, in the present study we tested whether items specifically pertaining to the experiential aspects of religion (i.e., mystical experiences that are typically characterized by a loss of sense of space and time) are related to a reduced volume of temporal brain regions, most notably the hippocampus (Owen et al., 2011) and the right middle temporal gyrus (Chan et al., 2009).

Thirdly, we tested whether an increased or decreased volume of gray matter in the bilateral SPL and inferior parietal lobes (IPL) is associated with a stronger religiosity and a higher proneness to having had a mystical‐like experience. This hypothesis partly follows from the initial work by Newberg by using functional neuroimaging data to establish the neural correlates of peak meditative experiences (Newberg, Alavi et al., 2001; Newberg & Iversen, 2003). He found that peak experiences of absolute unity are associated with a reduced blood flow to the superior parietal lobes and an increased activation of prefrontal areas, which he interpreted as being associated with a stronger focused attention. Other studies have used neuropsychological assessment techniques as an indirect proxy for superior parietal lobe functioning to establish a relationship between parietal lobe atrophy and religiosity (Johnstone & Glass, 2008; Johnstone et al., 2012, 2014; Urgesi et al., 2010). These studies indicate that a reduced activation or an impaired functioning of the parietal lobes (including the bilateral SPL and the IPL) is associated with a higher sensitivity for having spiritual experiences and increased religiosity. The supposed underlying mechanism is that the parietal lobes support a process of multi‐sensory integration and are at the basis of bodily self‐awareness (Blanke, 2012). A disruption of this process could result in changes in self‐awareness, for example, as observed during self‐transcendent and out‐of‐body experiences, as has been frequently observed in the neuropsychological literature (Blanke, Slater, & Serino, 2015). Only a few neuroanatomical studies have been conducted on the relationship between parietal lobe volume and mystical experience. Damage to the inferior parietal cortex has been associated with an increase in the personality trait of self‐transcendence in a group of 48 patients undergoing neurosurgery (Urgesi et al., 2010). This finding fits well with other studies on “religion‐by‐proxy” phenomena, such as the feeling of a presence, that have also been associated with damage to the inferior parietal lobe (for review, see: Blanke et al., 2015).

On the other hand, several studies also indicate that an increased volume of the parietal lobes is positively associated with religion and spirituality. One study, using data from 103 participants, found that increased importance of religiosity was associated with an increased volume of the left and right parietal cortices as well as the left precuneus (Miller et al., 2014). A different study showed that an increased IPL volume was associated with higher ratings of spirituality in a sample of 80 healthy participants (Van Schuerbeek et al., 2011). Also, doubting God's existence has been associated with a reduced volume of the right precuneus (Kapogiannis, Barbey, Su, Krueger et al., 2009)—although the sample size of this study was small. Thus, the relation between parietal lobe volume and religiosity and mystical experience is mixed. Therefore, we tested a direction‐unspecific hypothesis, by testing the relation between religious beliefs and mystical experiences in relation to either an increase or a decrease volume of the inferior parietal lobe.

We note that our theoretical predictions were quite generic and that the directionality of the expected effects is open to discussion. Still, we argue that—if there is any value in the neurocognitive mechanisms outlined above—this should have become visible in the present analysis, which could also serve to make more fine‐grained predictions for future studies. We are well aware that by relating religiosity to differences in gray matter volume, we somehow regress to the highly controversial phrenology approach (Jones, Alfaro‐Almagro, & Jbabdi, 2018). Rather than focusing on structural brain differences, it might make more sense to use network measures of brain activity and interaction between different brain regions, such as functional connectivity (Van Den Heuvel & Pol, 2010). We are very much in favor of using these techniques in association with religion and spirituality measures—and we definitely intend to use them in future studies. But our primary aim here was to establish the (absence of the) relation between religiosity and structural brain differences at a level of methodological and statistical rigor that we hope will set a new standard for future studies.

Thus, the specific hypotheses that we set out to test were the following: (a) a stronger acceptance of general religious beliefs is associated with a reduced volume of the bilateral orbitofrontal cortex; (b) a higher prevalence of mystical experiences is associated with a reduced volume of the right middle temporal gyrus and the hippocampus; (c) a higher prevalence of religious beliefs and mystical experiences is associated with an altered volume of the bilateral IPL. To test these predictions, we estimated gray matter volume throughout the entire brain using VBM and subsequently run both confirmatory ROI analyses of the relation between ROI‐average gray matter volume and religiosity and mystical experiences as well as a whole‐brain analysis of the relation between voxel‐wise gray matter volume and religiosity. The VBM procedure we used includes standard processing steps of the T1‐weighted scans, including bias‐correction, skullstripping, segregation of gray and white matter, non‐linear normalization to standard MNI space, and a Jacobian modulation step to correct for local expansion (or contraction) due to the non‐linear component of the spatial transformation (Douaud et al., 2007). The ROIs were defined using the Harvard–Oxford (sub)cortical probabilistic atlas (Craddock, James, Holtzheimer, Hu, & Mayberg, 2012; for more details on the ROI definition, see the Methods section).

The reason for doing ROI analyses on prespecified regions of interest was to obtain a high‐powered confirmatory test of the hypotheses derived from the literature. Typically, more restricted ROI analyses (relative to whole‐brain, voxel‐wise analyses) increase the statistical power to detect a potential effect (Cremers et al., 2017). Conducting confirmatory ROI analyses also allowed us to use Bayesian statistics on ROI‐average gray matter volume estimates, which provides the opportunity to quantify the relative evidence for the presence or absence of a relationship between religiosity and gray matter volume, which is not possible in the context of whole‐brain analyses because no standard software packages for VBM analyses offer Bayesian statistical tests. The ROI analyses focused on the following hypotheses which were primarily derived from the structural brain imaging studies (i.e., rather than the functional studies) discussed above: (a) a stronger acceptance of general religious beliefs is associated with a reduced volume of the orbitofrontal cortex; (b) a higher prevalence of mystical experiences is associated with a reduced volume of the right middle temporal gyrus and the hippocampus; (c) a higher prevalence of mystical experiences is associated with an altered volume of the inferior parietal lobe.

Next to conducting ROI analyses of prespecified brain regions forwarded by the literature, we also conducted a whole‐brain, voxel‐wise analysis. We believe this type of analysis is warranted given the quite unspecific nature of our hypotheses (e.g., next to the orbitofrontal lobe, other prefrontal areas such as the DLPFC have also been implicated in religiosity).

## 2 METHODS

### 2.1 Overview

**Figure 1:**
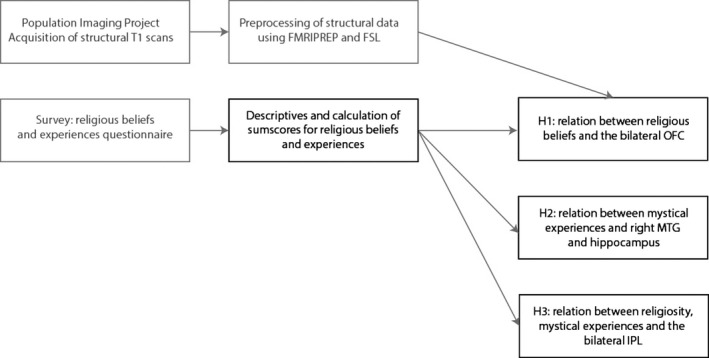
Overview of data acquisition and analysis strategy. Boxes marked in gray had already been completed prior to commencing this registered report. Boxes marked in black represent the analysis plan that was used for the present study [Colour figure can be viewed at wileyonlinelibrary.com]

An overview of the data collection and analysis procedure is presented in Figure 1. The data collection was already completed before the start of this project, and the structural MRI data have been checked visually using established quality metrics using the MRIQC tool (Esteban et al., 2017a; version 0.10.3) and preprocessed using FMRIPREP (Esteban et al., 2017b; version 1.0.15). For the present project, we analyzed the religiosity data to test the specific hypotheses by conducting an ROI and whole‐brain VBM analysis, focusing on the relation with religiosity and with mystical experiences.

### 2.2 Participants

Participants were recruited at the University of Amsterdam and consisted of students. In total 244 participants were tested, but 33 participants could not be used for the final analysis because of incomplete (MRI or behavioral) data or scanner artifacts (dropout rate = 8.2%), yielding a total sample size of *N *=* *211. The age range for participants was 20–28 years (mean = 24.18, *SD* = 1.92). The sample for this study consisted of 118 female participants and 93 male participants. All participants provided written informed consent before participating in the study and the experimental procedure was approved by the local ethics committee at the Psychology Department at the University of Amsterdam.

### 2.3 Outcome neutral criterion

As an outcome neutral criterion, we used the effect of (self‐reported) gender on gray matter volume in a separate VBM analysis. It is well established that there are structural differences in local and global gray matter structure between the brains of men of women (Good et al., 2001; Smith, Chebrolu, Wekstein, Schmitt, & Markesbery, 2007). Note that multivariate predictive analyses of the same VBM data have already shown that gender can be “decoded” from whole‐brain patterns of gray matter volume (Snoek, Miletic, & Scholte, 2018). While this multivariate analysis is different than the intended univariate analysis for this outcome neutral criterion, we believe that it demonstrates the validity of the proposed neutral criterion. By testing the main effect of gender on gray matter volume (by using a whole‐brain, voxel‐wise analysis on the same VBM data that was used for the religiosity analysis), we were thus able to show that our data are suitable for the intended main analysis. We expected to find widespread gender differences in gray matter volume throughout the brain (see e.g., Takahashi, Ishii, Kakigi, & Yokoyama, 2011).

### 2.4 Power analysis

In this project, we first conducted a set of ROI analyses based on prespecified brain areas that have been implicated in religious beliefs and mystical experiences. Next, given the rather broad and unspecific nature of the suggestions in the literature, we also conducted a whole‐brain analysis (of which the results were corrected for multiple comparisons).

There are multiple ways in which a power analysis could be conducted. Here, we based the estimated effect size on the reported effects in neuroanatomical studies on religiosity and mystical experience (Cristofori et al., 2016; Hayward et al., 2011; Owen et al., 2011; Van Schuerbeek et al., 2011). Although these papers did not always provide sufficient detail to obtain a standardized effect size, overall the reported effects were small, that is, *β*‐values ranged from .12 to .22 (Hayward et al., 2011; Owen et al., 2011), and *η* ^2^ ranged from .01 to .07 (Cristofori et al., 2016; Zhong et al., 2017). Assuming a small effect size for our analysis of *r *=* *.20, a sample size of *N* = 224 and an alpha‐level of *p *<* *.05, the achieved power of our analysis was 1 − *β* = .92, meaning that there was 92% chance of correctly rejecting the null hypothesis that there was no relation between religiosity and brain volume (note, however, that strictly speaking our intended Bayesian analyses do not employ the null‐hypothesis testing framework assumed by power analyses). This criterion exceeds the critical threshold of at least 80% statistical power (Cohen, 1992), and we note that our sample size far exceeds that of most previous studies on this topic. Thereby, we aimed to provide a more precise estimate of the effect size regarding the relation between structural brain differences and religiosity.

### 2.5 Population imaging project

The data for this study were collected as part of the Population Imaging of Psychology project (PIoP1), which was conducted at the Spinoza Center for Neuroimaging at the University of Amsterdam. The aim of the PIoP was to offer researchers the opportunity to collect brain imaging data from a large sample of participants (intended *N *=* *250), in association with their individual difference measure of interest. The data were collected between May 2015 and April 2016. The MRI data have been preprocessed by LS and have been used already for a project to identify multivariate structural brain differences in association with gender (Snoek et al., 2018). The behavioral data (i.e., religiosity questionnaires) have been acquired by MvE but had not been subjected to any analysis so far. Both authors have confirmed that the MRI data have not been associated in any way to the behavioral data and that all hypotheses and the processing pipeline were developed and defined prior to data inspection.

Standard MRI measurements that were collected for each participant included a structural T1‐weighted scan, task‐free resting state fMRI (6 min), a diffusion‐weighted imaging (DWI) scan, and different functional localizer scans that were collected using gradient‐echo EPI sequences, including the Gender Stroop Task, an emotional matching task (Hariri, Bookheimer, & Mazziotta, 2000), a working memory task (Pessoa, Gutierrez, Bandettini, & Ungerleider, 2002) and the anticipation of negative emotional vs. neutral scenes (Oosterwijk, 2017). In addition, for each participant background demographic variables were recorded (gender, age, socio‐economic status), as well as the NEO‐FFI personality questionnaire (Costa & MacCrae, 1992) and an intelligence test (Raven's matrices; Raven, 2000). For the present study, we included measures related to religiosity and mystical experiences (for description, see below).

### 2.6 Religiosity measures

For this study, we selected 7 items to measure religiosity, which were completed using a 5‐point Likert scale ranging from 1 = not at all to 5 = very much (see Table 1). Six out of these seven questions were directly based on the items that are used to measure religiosity in the world value survey (Freese, 2004): three items assessed people's religious beliefs (i.e., religiosity, belief in God, belief in afterlife), two items assessed the importance of people's faith for their lives, and two items assessed participants’ religious practices (i.e., prayer and church visit). Although these questions are not part of a standardized and validated scale to measure religiosity, the face validity of the items is high (e.g., church visit and prayer refer to easily identifiable behaviors) and the construct validity can be further guaranteed based on other items that were included. Next to the religiosity items, we also asked whether participants considered themselves to be a member of a church or a religious organization, and if so whether they could indicate their religious denomination (open response). In this way, we could establish whether participants who indicate religious membership indeed scored higher on the religiosity questions.

**Table 1:** Items included to measure religiosity. All items were completed by using a 5‐point scale ranging from 1 = not at all to 5 = very much

To what extent do you consider yourself to be religious?
To what extent do you believe in God or a supernatural being?
To what extent do you believe in life after death?
My faith is important to me
My faith affects my thinking and practice in daily life
I pray daily
I visit a church or religious meeting on a weekly basis

We also asked three questions about the religious beliefs (religiosity) and practices (church visit and lifestyle) of the participants’ parents. Previous studies have shown that one's parents’ religiosity, specifically the extent to which they show credibility enhancing displays of their beliefs (e.g., wearing religious clothing, going to religious meetings), is a strong predictor of endorsing religious beliefs (Lanman & Buhrmester, 2017; Maij, van Harreveld et al., 2017). As such, determining one's parents’ religiosity provides a good way to further establish the construct validity of our religiosity scale. Thus, for the VBM analysis we used the seven religiosity questions as presented in Table 1 as predictor variables.

In addition, we included 6 items to measure mystical‐like experiences, which were completed using a 5‐point Likert scale ranging from “1 = not at all” to “5 = very much” (see Table 2). These items were items related to mystical experiences from the Tellegen absorption scale (Tellegen & Atkinson, 1974) and items from the mysticism scale (Hood, 1975). In several studies, it has been found that one's scores on these items are strongly predictive of self‐induced mystical experiences (van Elk, 2015; Maij & van Elk, 2018; Maij, van Elk, & Schjoedt, 2017), self‐transcendent feelings of awe (van Elk, Karinen, Specker, Stamkou, & Baas, 2016) and hearing the voice of God (Luhrmann, 2011; Luhrmann, Nusbaum, & Thisted, 2013). Accordingly, for the VBM analysis of mystical experiences, we used the sumscore of the six items in Table 2 as predictor variables. Next to the questions that were included in the present analysis, we also asked questions about the participants’ spirituality, paranormal beliefs, conspiracy beliefs, and their level of absorption.

**Table 2:** Items included to measure mystical experiences. All items were completed by using a 5‐point scale ranging from 1 = not at all to 5 = very much

I have had an experience which was both timeless and spaceless
I have had an experience in which something greater than myself seemed to absorb me
I have had an experience in which I felt myself to be absorbed as one with all things
I have had an experience, of which I was incapable of being expressed in words
I have had an experience in which I realized the oneness of myself with all things
I think I really know what some people mean when they talk about mystical experiences

It could well be that average ratings of religiosity and mystical experiences are non‐normally distributed, as data were mainly collected from secularized students. However, we note that this is not an issue for the statistical assumptions of the analyses on the VBM data, which are based on the general linear model (GLM) that assumes normality of the model's residuals, but not normality of its predictors. Moreover, given results from earlier studies (see for instance: van Elk, Rutjens, van der Pligt, & Van Harreveld, 2016) and the fact that this study's sample consistent of university students, relatively few participants scored high on religiosity and mystical experiences. However, while potential low variance in the predictor‐of‐interest (i.e., religiosity and mystical experiences) may reduce power (Poldrack, Mumford, & Nichols, 2011), this study's relatively large sample size compensates for this statistical inefficiency.

### 2.7 VBM processing pipeline

The T1‐weighted scans with a voxel size of 1.0 × 1.0 × 1.0 mm were acquired using 3D fast field echo (TR: 8.1 ms, TE: 3.7 ms, flip angle: 8°, FOV: 240 × 188 mm, 220 slices). The T1‐weighted anatomical scan was bias‐corrected, skullstripped and segmented using the FMRIPREP package (version 1.0.0; Esteban et al., 2017b)—a Nipype (Gorgolewski et al., 2011) based tool. Each T1 weighted volume was corrected for bias field using N4BiasFieldCorrection (v2.1.0; Tustison et al., 2010) and skullstripped using antsBrainExtraction.sh v2.1.0 (using the OASIS template). Three tissue classes were extracted from T1w images using FSL FAST (v5.0.9; Jenkinson, 2003). From here on, we followed the “FSL‐VBM” protocol (Douaud et al., 2007) from the FSL software package (version 5.0.9; Smith et al., 2004). The gray matter maps were registered to the MNI 152 standard space using non‐linear registration (Andersson, Jenkinson, & Smith, 2007). The resulting images were averaged and flipped along the *x*‐axis to create a left‐right symmetric, study‐specific gray matter template. Second, all native gray matter images were non‐linearly registered to this study‐specific template and “modulated” to correct for local expansion (or contraction) due to the non‐linear component of the spatial transformation. The modulated gray matter images were then smoothed with an isotropic Gaussian kernel with a sigma of 3 mm.

We used a volume‐based approach rather than a surface‐based approach, to preserve consistency with previous studies on this topic (Cristofori et al., 2016; Kapogiannis, Barbey, Su, Krueger et al., 2009; Van Schuerbeek et al., 2011).

### 2.8 ROI analyses

The ROI analyses focused on the following hypotheses: (a) a stronger acceptance of general religious beliefs is associated with a reduced volume of the orbitofrontal cortex; (b) a higher prevalence of mystical experiences is associated with a reduced volume of the right middle temporal gyrus and the hippocampus; (c) a higher prevalence of mystical experiences and religiosity is associated with an altered volume of the bilateral IPL (which we define as the combination of the angular gyrus and the supramarginal gyrus). ROIs for these brain areas were identified using the probabilistic Harvard–Oxford (sub)cortical atlas (see Table 3). To create a binary mask, we thresholded the probabilistic ROIs at 0 (i.e., any voxel with a non‐zero probability of belonging to that brain area were included in the binary mask). For each participant, we averaged the voxel‐wise gray matter volume estimates within each ROI separately, which served as the dependent measure for our ROI analyses.

**Table 3:** Regions of interest for the ROI analysis to assess the relation between religious beliefs and mystical experiences and gray matter volume

Religious beliefs ROIs	Sub‐regions (from Harvard‐Oxford atlas)
(1) Orbitofrontal cortex	—
(3) Bilateral inferior parietal lobes	Bilateral angular gyrus Bilateral supramarginal gyrus
Mystical experiences ROIs	
(1) Hippocampus	Bilateral hippocampus
(2) Right middle temporal gyrus	Right anterior MTL Right posterior MTL
(3) Bilateral inferior parietal lobes	Bilateral angular gyrus Bilateral supramarginal gyrus

For our ROI analyses, we used a Bayesian ANCOVA model. We used a Bayesian ANCOVA instead of Bayesian regression because the statistical program we used, JASP (Marsman & Wagenmakers, 2017; version 0.9.2), does not allow for categorical independent variables in their Bayesian regression implementation, which prevents us from including gender as independent (“nuisance”) variable. Next to gender, we included age and intelligence (operationalized as the sumscore on the Raven's matrices test) as “nuisance” variables. The rationale for including these measures as dummy variables in our analysis is to control for the potential confound that any religiosity effect might be driven by other individual differences that are known to be associated with religiosity: typically females are more religious than males (Miller & Hoffmann, 1995); older participants tend to be more religious (Argue, Johnson, & White, 1999); and people scoring high on intelligence are on average less religious (Zuckerman, Silberman, & Hall, 2013).

As our main independent variables of interest, we included our two religiosity measures of interest (i.e., religiosity and mystical experiences). We reported the Bayes factors for the model including the main independent variables of interest compared to the null model containing the nuisance variables (gender, level of education, intelligence, and age). We ran the Bayesian ANCOVA analysis for each ROI separately.

### 2.9 Whole‐brain analysis

For the whole‐brain analysis, we used a non‐parametric, permutation‐based (frequentist) GLM (using 10,000 random permutations) with threshold‐free cluster enhancement (TFCE; Smith & Nichols, 2009) using FSL's “randomize” tool. Using TFCE‐based statistics instead of regular cluster‐based statistics allows us to draw inferences on the voxel‐level, which affords more detailed conclusions of the location of potential significant correlations with religiosity (Smith & Nichols, 2009). The TFCE‐values were corrected for multiple comparisons using the maximum statistic approach in which voxels were only be considered significant if the observed TFCE test statistic falls within the highest or lowest 2.5th percentile of the distribution of the permuted maximum statistic values (i.e., voxel‐wise *α* = .025).

Similar to the ROI analyses, we included gender, age and intelligence as covariates in our whole‐brain analysis. For this analysis, we specified two contrasts, one for each main independent variable of interest, which represent tests of whether regression coefficients differ from zero. Because the literature reports both positive and negative correlations between religiosity measures and gray matter volume, we tested the contrasts in both directions and adjust the significance level accordingly (i.e., use an alpha of 0.025 instead of the conventional 0.05; Chen et al., 2019). Thresholded (i.e., significant) results were visualized using the MNI152 (2 mm) template with different colors indicating positive versus negative effects.

To include religiosity and mystical experiences as regressors in our model, first for each scale we calculated the reliability by using Cronbach's *α*. Next, the sumscores for each scale were calculated, which were used as predictors in the statistical model (from both the ROI analyses and whole‐brain analysis).

## 3 RESULTS

### 3.1 Data and code availability

Most data and all code for this study are deposited in publicly available online repositories. All analysis code and code to reproduce the figures of this manuscript are available from the project's GitHub repository: https://github.com/lukassnoek/ReligiosityVBM. This repository also contains a csv‐file with the data to reproduce the ROI analyses (i.e., the ROI‐average gray matter volume, nuisance variables and religious belief/mystical experience variables). Unthresholded brain maps from the whole‐brain analysis of both the outcome neutral test and main analysis can be viewed and downloaded from this project's Neurovault repository: https://identifiers.org/neurovault.collection:5380. Lastly, the project was preregistered on the open‐science framework (OSF) at https://osf.io/qzkmh/.

Below, we describe the results from both the outcome neutral analyses and the main analyses. The unthresholded brain maps from the whole‐brain analyses for both the outcome neutral and main analyses can be found in this study's neurovault repository and the data for the ROI analyses (i.e., the ROI‐average gray matter volume and covariates) can be found in this study's GitHub repository (see Data and Code availability).

### 3.2 Deviations from preregistration

Although we planned to use data from *N* = 224 participants in our analysis, in the end we were only able to include data from *N* = 211 participants. This was the result of participants that were missing either MRI data or religiosity data.

### 3.3 Descriptive statistics

For the final analysis, 211 participants (118 females) were retained. The descriptive variables, including religiosity and personality characteristics, are presented in Tables 4 and 5. Both the religiosity and the mystical experience scale showed a good reliability, Cronbach's *α* = .880 and *α* = .877, respectively. As can be seen in the correlation table, religiosity was negatively correlated with intelligence, and mystical experiences were positively correlated to religiosity—although overall correlations were small. As expected, participants who indicated to be a member of a church scored higher on the religiosity scale (mean = 3.38, *SE* = 0.30) than those who did not (mean = 1.73, *SE* = 0.05), *t*(209) = 8.52, *p* < .001.

**Table 4:** Descriptive statistics for the participants included in the VBM analysis (*N* = 211)

	Age	Raven	Religiosity	Mystical	A	C	E	N	O
Mean	24.18	24.47	1.725	2.475	43.93	43.27	44.47	30.79	41.64
Std. deviation	1.924	4.997	0.8093	1.139	5.012	6.900	5.257	7.527	6.072
Minimum	20.00	3.000	1.000	1.000	27.00	22.00	31.00	13.00	28.00
Maximum	28.00	35.00	5.000	5.000	56.00	59.00	56.00	58.00	58.00

Abbreviations: A, agreeableness; C, conscientiousness; E, extraversion; N, neuroticism; O, openness to experience (scores on the NFFI personality questionnaire).

**Table 5:** Correlations between the different variables included in this study

	Age	Raven	Religiosity	Mystical	A	C	E	N	O
Age	—								
Raven	−0.001	—							
Religiosity	0.013	−0.141*	—						
Mystical	−0.107	−0.032	0.232***	—					
A	−0.040	0.102	0.095	0.003	—				
C	0.044	−0.071	0.086	0.107	0.198**	—			
E	0.059	0.012	0.042	−0.019	0.207**	0.121	—		
N	0.070	−0.115	0.130	0.044	0.002	−0.209**	−0.297***	—	
O	−0.022	−0.021	0.061	0.029	0.093	−0.176*	−0.059	0.201**	—

Abbreviations: A, agreeableness; C, conscientiousness; E, extraversion; N, neuroticism; O, openness to experience (scores on the NFFI personality questionnaire).

*p < .05.

**p < .01.

***p < .001.

Females in our study were slightly older than males (mean = 24.53, *SE* = 0.16, and mean = 23.74, *SE* = 0.21, respectively), *t*(209) = 2.99, *p *=* *.003. There was no effect of gender on religiosity, but females tended to score lower on mystical experiences (mean = 2.21, *SE* = 0.10) than males (mean = 2.81, *SE* = 0.12), *t*(209) = −3.90, *p *<* *.001. No differences were found between males and females on the NNFI personality traits, *t*(209) < 1.36, *p* > .174.

### 3.4 Outcome neutral results

**Figure 2:**
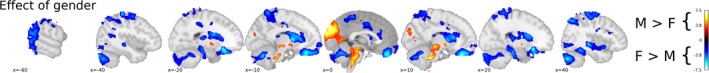
Whole‐brain significant (*ɑ* = 0.025) voxel‐wise *t‐*statistics of the effect of gender computed with a (non‐parametric) general linear model on the threshold‐free cluster enhancement‐transformed and thresholded voxel‐based morphometry data. Red‐yellow voxels represent a significantly higher local gray matter volume for male than for female participants, while blue voxels represent a significantly higher local gray matter volume for female than for male participants. Unthresholded statistical brain maps (*t*‐values and 1 − *p* maps) can be viewed at and downloaded from https://identifiers.org/neurovault.collection:5380 [Colour figure can be viewed at http://wileyonlinelibrary.com]

For the outcome neutral test, we investigated the effect of (self‐reported) gender on gray matter volume in a whole‐brain non‐parametric voxel‐wise analysis using the *randomize* function from the FSL software package. In Figure 2, we plot the significantly different voxels (two‐sided *t* test) resulting from this analysis.

### 3.5 ROI analyses

**Figure 3:**
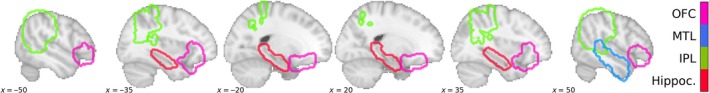
Outline of region‐of‐interests (ROIs) used in this study (Hippoc., hippocampus; IPL, inferior parietal lobe; MTL, mediotemporal lobe; OFC, orbitofrontal cortex). All ROIs were bilateral, except for the (right hemisphere) MTL [Colour figure can be viewed at http://wileyonlinelibrary.com]

Our ROI analyses for *religious belief* were done on the bilateral OFC and the bilateral IPL, while the ROI analyses for *mystical experience* were done on the bilateral hippocampus, right MTL and bilateral IPL (see Figure 3).

The ROI analyses are based on average gray matter volume within a particular ROI. We used the Bayesian ANCOVA module in the statistical software package “JASP” for our ROI analyses (Love et al., 2015; Morey & Rouder, 2015; Rouder, Morey, Speckman, & Province, 2012). In the Bayesian ANCOVA analysis, we used the ROI‐average gray matter volume as dependent variable, gender as fixed factor, and intelligence, age, and religious belief or mystical experience as covariates. The variables gender, intelligence and age were added to the “null model,” which we compared to our “religious belief model,” in which we include the religious belief covariate or “mystical experience model,” in which we include the mystical experience covariate.

#### 3.5.1 Religious belief

For both the OFC and IPL, there was more evidence for the null model than for the “religious belief” model, with Bayes factors (BF_10_) of 0.357 (OFC) and 0.414 (IPL), suggesting that the data under the null model is more plausible than under the religious belief model.

#### 3.5.2 Mystical experience

Similar to the religious belief analyses, for all three ROIs (IPL, rMTL and hippocampus) there was weak evidence for the *null model*, with Bayes factors (BF_10_) of 0.283 (IPL), 0.357 (rMTL) and 0.328 (hippocampus), again suggesting that the data under the null model is more plausible than under the mystical experience model.

### 3.6 Whole‐brain analysis

**Figure 4:**
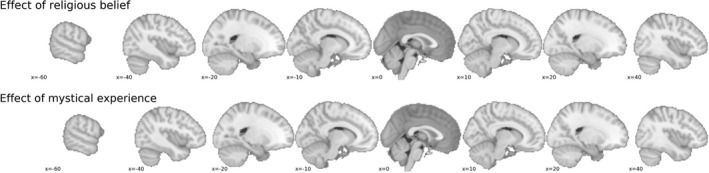
Whole‐brain results of religious belief and mystical experience contrasts. After multiple comparison correct, no voxels showed a significant difference from zero. Unthresholded statistical brain maps (*t*‐values and 1 − *p* maps) can be viewed at and downloaded from https://identifiers.org/neurovault.collection:5380 [Colour figure can be viewed at wileyonlinelibrary.com]

In addition to the ROI analyses of religious belief and mystical experience, we also conducted a whole‐brain voxel‐wise analysis with religious belief and mystical experience as covariates (with identical settings as the outcome neutral whole‐brain analysis). We used a significance level of 0.025 as we conducted a two‐sided test (i.e., we tested both for positive and negative associations of our covariates of interest with the VBM data; cf., Chen et al., 2019). As can be seen in Figure 4, no voxels were found to be significant after multiple comparison correction. Unthresholded whole‐brain maps can be found in the neurovault repository belonging to this study.

### 3.7 Exploratory results

**Figure 5:**
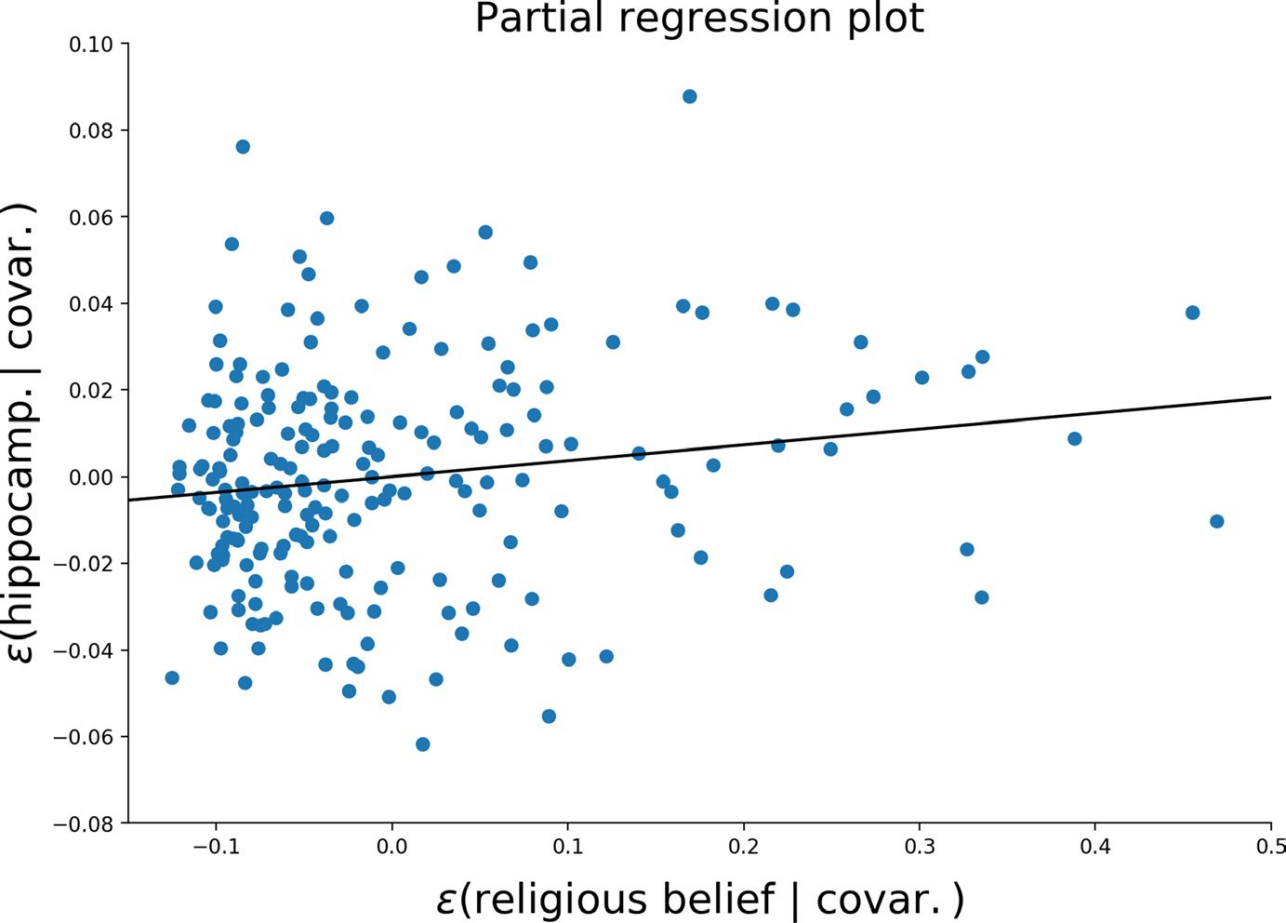
The regression line describes the effect of religious belief on hippocampal gray matter volume after partialling out the effects of gender, intelligence and age, indicating a Bayes factor (BF_10_) of 3.512 in favor of the model including religious belief. The partial regression analysis was performed using the *statsmodels* Python package [Colour figure can be viewed at http://wileyonlinelibrary.com]

In addition to the preregistered analyses, in an exploratory analysis we found hippocampus gray matter volume was positively associated with religious belief (after adjusting for age, intelligence and gender), as indicated by a Bayes factor (BF_10_) of 3.512 in favor of the model including religious belief. Although this Bayes factor suggests a moderate amount of evidence for the observed effect (Jeffreys, 1961), we stress that the reader should interpret this effect with care as this analysis was not preregistered. To aid the interpretation of the strength of the effect, Figure 5 shows a partial (frequentist) regression plot, showing the effect of religiosity on hippocampal gray matter volume after partialling out the effects of age, intelligence and gender.

## 4 DISCUSSION

In this registered report, we investigated whether religiosity and mystical experiences were associated with structural brain differences in gray matter volume. By using an outcome neutral criterion, we were able to show the validity of our experimental and analytical approach, by identifying clear gender differences in gray matter volume between men and women (Takahashi et al., 2011). However, we did not observe structural brain differences in association with self‐reported religiosity or mystical experiences, neither using an ROI analysis, nor using a whole‐brain analysis. Overall, we observed moderate evidence for the null model according to which gray matter volume in the OFC, the bilateral IPL, the rMTL and the hippocampus are best explained by gender, age and intelligence, rather than religiosity or mystical experiences.

These findings cast new light on the claim that religion is hardwired in the brain. Many previous studies in the field of the neuroscience of religion have suffered from methodological problems, such as the lack of experimental control, problems with ecological validity and low statistical power (Schjødt & van Elk, 2019). The current replication study comprised a relatively large sample and we used well‐validated measures of religiosity and mysticism, thereby overcoming the limitations of previous research. Based on a thorough literature review, we also used an ROI‐based analysis, resulting in a relatively high statistical power. Still, the outcomes were not promising: religiosity and mystical experiences were not consistently related to gray matter volume differences. We note that in our exploratory analysis a positive correlation was found between hippocampal gray matter volume and religiosity. This finding needs to be interpreted with caution as it was not preregistered and the correlation is also contrary to the effects that have been observed in earlier studies, indicating that hippocampal atrophy was related to an increased religiosity, that is, a negative correlation between hippocampal gray matter volume and religiosity (Chan et al., 2009; Owen et al., 2011). Still, a future independent replication study could take this unexpected finding into account, by conducting a confirmatory ROI analysis of this relationship.

The absence of a clear and consistent relation between religiosity and structural brain differences may not appear surprising in the light of the recent replication crisis that has haunted psychology and neuroscience as well (Zwaan, Etz, Lucas, & Donnellan, 2017). Previous replications attempts have shown that correlations between structural brain properties and behavior and personality measures in general are notoriously difficult to replicate (Boekel et al., 2015; Melonakos et al., 2011). The field of neuroscience is plagued with many low‐powered studies and accordingly the literature abounds with many false‐positive findings, resulting in an overall inconsistent and scattered pattern of results (Button et al., 2013). Another problem related with identifying the structural brain correlates of religiosity is that other confounding factors tend to covary with religion, such as gender, age, schizotypy but also mental and physical health (e.g., living a healthier lifestyle by adhering to one's religious prescriptions; cf., Maltby, Garner, Lewis, & Day, 2000; Miller & Hoffmann, 1995; Stavrova, Fetchenhauer, & Schlösser, 2013). These factors in turn also directly have an effect on gray matter volume (Goodkind et al., 2015; Modinos et al., 2010), thereby further obscuring an eventual effect.

On a more positive note, a promising alternative to studying structural brain differences is the use of multivariate pattern recognition (Calhoun, Lawrie, Mourao‐Miranda, & Stephan, 2017) and network analysis techniques (Sporns, 2014). These methods provide an increased sensitivity, assuming that confounds are properly controlled for (Snoek, Miletić, & Scholte, 2019), because they allow identifying multidimensional spatially distributed representations, which is beyond the reach of classic univariate approaches (Jimura & Poldrack, 2012). Relatedly, as already outlined in the Introduction, several functional neurocognitive mechanisms have been proposed to underlie a general propensity for religiosity and religious experiences, such as for instance a reduced error monitoring mechanism (van Elk & Aleman, 2017). Putting these ideas to the test would require setting up carefully designed functional neuroimaging studies. These would need to do justice to both the requirement to study authentic religious beliefs and practices, while also providing sufficient experimental control (Schjødt & van Elk, 2019). We note that we currently have two studies underway in line with this approach: in one study, we assess the effects of source credibility in believers vs. non‐believers (Schjoedt et al., 2011), and while in the other, we assess the relationship between neurocognitive conflict detection in a Stroop task and religiosity (Hoogeveen, Snoek & van Elk, in prep.). An alternative and complementary approach is to deconstruct religion in its constitutive components, such as rituals, morality and belief in minimally counterintuitive concepts (McKay & Whitehouse, 2015). Each of these topics could be related to the extant literature in social and cognitive neuroscience.

## 5 CONCLUSION

In this study, we found no evidence that religiosity is associated with a reduced volume of the orbito‐frontal cortex and changes in the structure of the bilateral inferior parietal lobes. Neither did we find support for the notion that mystical experiences are associated with a reduced volume of the hippocampus, the right middle temporal gyrus or with the inferior parietal lobes. A whole‐brain analysis furthermore indicated that no structural brain differences were found in association with religiosity and mystical experiences. The search for the neural correlates of religious beliefs and experiences should therefore probably shift focus from studying structural brain differences, to a functional and multivariate approach.

## CONFLICT OF INTEREST

The authors declare to have no conflict of interest.

## AUTHOR CONTRIBUTIONS

MvE designed the study; MvE & LS wrote the RR; LS supervised data collection; LS analyzed the data with input from MvE.

## Data Availability

All analysis code and code to reproduce the figures of this manuscript are available from the project's GitHub repository: https://github.com/lukassnoek/ReligiosityVBM. This repository also contains a csv‐file with the data to reproduce the ROI analyses (i.e., the ROI‐average gray matter volume, nuisance variables and religious belief/mystical experience variables). Unthresholded brain maps from the whole‐brain analysis of both the outcome neutral test and main analysis can be viewed and downloaded from this project's Neurovault repository: https://identifiers.org/neurovault.collection:5380. The project was preregistered on the open‐science framework (OSF) at https://osf.io/qzkmh/.
